# Notes on the genus *Xenocerogria* (Coleoptera, Tenebrionidae, Lagriini) from China

**DOI:** 10.3897/zookeys.451.8478

**Published:** 2014-11-03

**Authors:** Yong Zhou, Ottó Merkl, Bin Chen

**Affiliations:** 1 Institute of Entomology and Molecular Biology, College of Life Sciences, Chongqing Normal University, Chongqing 401331, P.R. China; 2 Hungarian Natural History Museum, H-1088 Budapest, Baross utca 13, Hungary

**Keywords:** China, Lagriinae, lectotype designation, new synonym, redescription, Tenebrionidae, *Xenocerogria*

## Abstract

Three species of the genus *Xenocerogria* Merkl, 2007 have been recorded in China, *Xenocerogria feai* (Borchmann, 1911), *Xenocerogria ignota* (Borchmann, 1941) and *Xenocerogria ruficollis* (Borchmann, 1912). *Xenocera xanthisma* Chen, 2002 is proposed as a junior synonym of *Xenocerogria ruficollis*. Lectotype of *Xenocerogria ignota* is designated, and the species is transferred to the genus *Lagria* Fabricius, 1775. New Chinese province records of *Xenocerogria ruficollis* are provided.

## Introduction

*Xenocerogria* Merkl, 2007 is a small genus of Lagriini distributed in China, India and Southeast Asia. The generic name *Xenocerogria* was proposed by Merkl (2007) as a new replacement name for *Xenocera* Borchmann, 1936, preoccupied by *Xenocera* Broun, 1881 (Anobiidae).

The genus *Xenocera* was established by Borchmann (1936) with *Lagriocera feai* Borchmann, 1911 from “Carin Chebà, Burma” designed as its type species (“Gattungstype” in the original description). Simultaneously, Borchmann (1936) assigned further six species to the genus all originally described in *Lagriocera* Fairmaire, 1896. One of them, *Xenocera ruficolli* s was recorded from China. Borchmann (1941) then added *Xenocera ignota*, described from China, Fujian. Merkl (2007) recorded *Xenocerogria feai* as a species new to China (Yunnan). Chen (2002) described *Xenocera xanthisma*, also from China (Fujian and Hunan). In the present study, we found that *Xenocera xanthisma* is the junior synonym of *Xenocerogria ruficollis*. Therefore, there are eight known species of the genus, of which three species have distribution records in China.

No modern revision has been published for the genus *Xenocerogria*, only a checklist was provided by Merkl (2007). In the present paper, all the three Chinese species are redescribed. Based on the male described hereunder, *Xenocera ignota* is removed from the genus, and transferred to the large collective genus *Lagria* Fabricius, 1775.

## Material and methods

Photographs of the types of *Xenocera xanthisma* Chen, 2002 were taken by Leica M205A stereomicroscope; descriptions and measurements were performed under a stereomicroscope (Nikon SMZ1500), and photomicrographs of *Xenocerogria ruficollis* (Borchmann, 1912) were taken with a stereomicroscope (LEICA EZ4 HD) attached to a computer using Leica Application Suite version 2.1.0 software in Chongqing. Photographs of *Xenocerogria feai* and *Xenocerogria ignota* were taken with Nikon Coolpix 4500 digital camera attached to Leica MZ 125 stereomicroscope in Budapest. Label text of type specimens is cited verbatim.

The following abbreviations are used for institutions where specimens are deposited (curators responsible for loans in parentheses):

CCLT Private collection of Chi-Feng Lee, Taipei, Taiwan;

CKMT Private collection of Kimio Masumoto, Tokyo, Japan;

CMNH Carnegie Museum of Natural History, Section of Invertebrate Zoology, Pittsburgh, PA, USA (R. Davidson);

CQNU Chongqing Normal University, Chongqing, P. R. China (Bin Chen);

CSBC Private collection of Stanislav Bečvář, České Budějovice, Czech Republic;

DEI Deutsches Entomologisches Institut, Müncheberg, Germany (Lothar Zerche);

HNHM Hungarian Natural History Museum, Budapest, Hungary (Ottó Merkl);

MHBU Museum of Hebei University, Baoding, P. R. China (Guo-Dong Ren);

MHNG Muséum d’histoire naturelle, Geneva, Switzerland (Giulio Cuccodoro);

MSNG Museo Civico di Storia Naturale “Giacomo Doria”, Genova, Italy (Roberto Poggi);

NSMT National Science Museum (Natural History), Tokyo, Japan (Shuhei Nomura);

QCCC Private collection of Jian-Yue Qiu & Hao Xu, Chongqing, P. R. China;

SMNS Staatliches Museum für Naturkunde, Stuttgart, Germany (Wolfgang Schawaller);

SWU Southwest University, Chongqing, P. R. China (Li Chen);

ZFMK Zoologisches Forschungsinstitut und Museum Alexander Koenig, Bonn, Germany (Michael Schmitt).

## Taxonomy

### Xenocerogria

Taxon classificationAnimaliaColeopteraTenebrionidae

Merkl, 2007

Xenocera Borchmann, 1936: 116 (not Broun, 1881: 668). Type species: *Lagriocera feai* Borchmann, 1911, by original designation.
Xenocerogria Merkl, 2007: 269 (replacement name); Merkl 2008: 116.

#### Diagnosis.

The genus was thoroughly described by Borchmann (1936), so only the most important characteristics are mentioned here. **Male:** Body small, body length 5–10 mm. Head slightly rounded. Apical maxillary palpomere securiform and thick. Labrum with anterior margin more or less emarginate. Clypeus with anterior margin emarginate, exposing labroclypeal membrane. Frontoclypeal suture deep and arcuate. Antennae strong, antennomeres 9 and 10 strongly transverse, with produced anterolateral corner, antennomere 11 strongly elongate, as long as combined length of at least 4 preceding antennomeres, concave ventrally. Pronotum slightly convex, slightly narrower than head. Elytra about 2× as wide as pronotum, slightly broadened toward apex, without striae, with irregular, but evenly scattered punctation. Legs simple, moderately robust; femur not clavate, tibia nearly straight, without modifications. **Female:** Similar to male but larger and broader. Antennomere 11 shorter than combined length of 3 preceding antennomeres. Pronotum slightly wider than head. Elytra more broadened toward apex.

#### Distribution.

China, India, Burma, Java, Sumatra.

#### Remarks.

This genus is distinguished from other lagriine genera on the basis of the male antenna: antennomeres 9 and 10 are strongly transverse; the terminal antennomere is concave in ventral surface, with length equal to at least combined length of 4 preceding antennomeres (female with shorter terminal antennomere). This antennal structure is combined with unmodified male tibiae. These characteristics make this genus vaguely defined, and it is possibly an artificial assemblage of species not closely related to each other. Even Borchmann (1936) himself emphasized that one of the species, *Xenocerogria andrewesi* (Borchmann, 1925) is somewhat different from other species: antennae are more slender, only antennomere 10 is transverse, and pronotum of female with a wide longitudinal impression.

The situation is similar in most genera of the tribe Lagriini. Most of the species of the subtribe Lagriina were originally described as members of the genus *Lagria* Fabricius, 1775. Later, species with unusual (apomorphic) characters were transferred from *Lagria* to separate genera established mainly by Borchmann, for instance *Aulonogria* Borchmann, 1929, *Cerogria* Borchmann, 1909, *Neogria* Borchmann, 1911 and *Schevodera* Borchmann, 1936, just to mention a few from East and Southeast Asia. However, these genera are defined mostly by modifications of male antennomeres and tibiae. Females not associated with males are difficult or virtually impossible to separate at generic level. The remaining species were retained in the genus *Lagria*. In fact, modifications can frequently be observed on the male antennae and legs of species of *Lagria*, although these are not as prominent as in *Cerogria*, for instance. The genus *Lagria* itself, used as a dumping ground for more “simple” species is therefore still quite diverse, and most of the Oriental species are rather different from the type species, the western Palaearctic *Lagria hirta* (Linnaeus, 1758).

Removal of species with unique characters, creating a hardly treatable mass of less distinctive species in a large genus is common throughout the Coleoptera, including the family Tenebrionidae (see comments by Campbell 2014 and Schawaller 2014). A natural classification of the subtribe Lagriina would be achieved by study of (often unavailable) types of all described species supported by extensive molecular studies, but this must be an enormous undertaking.

#### Key to Chinese species presently and formerly assigned to *Xenocerogria*

**Table:** 

1(2)	Elytral pubescence short and completely decumbent. Male: antennomere 3 much longer than 1 and 2 combined, antennomeres 9 and 10 not transverse, antennomere 11 unmodified, much shorter than 3 preceding combined; inner side of hind tibia finely denticulate	***Lagria ignota* (Borchmann, 1941)**
2(1)	Elytral pubescence longer, semierect to erect. Male: antennomere 3 much shorter than 1 and 2 combined, antennomeres 9 and 10 transverse, antennomere 11 enlarged, concave, as long as at least 4 preceding combined; hind tibia without denticulation.	
3(4)	Elytral pubescence erect. Male: antennomere 11 as long as 4 preceding combined; antennomeres 5 to 10 with inner surface flattened, bordered with longitudinal carina. Female: length of elytra about 2× maximum width	***Xenocerogria feai* (Borchmann, 1911)**
4(3)	Elytral pubescence semierect. Male: antennomere 11 as long as 6 preceding combined; antennomeres 5 to 10 without flattened inner surface. Female: length of elytra about 3× maximum width	***Xenocerogria ruficollis* (Borchmann, 1912)**

### Xenocerogria ruficollis

Taxon classificationAnimaliaColeopteraTenebrionidae

(Borchmann, 1912)

Figs 1 –13

Lagriocera ruficollis Borchmann, 1912: 7 (type locality: China: Taiwan; type depository: DEI).
Lagriocera ruficollis : Borchmann 1916: 127 (China: Taiwan); Kôno 1929: 29 (China: Taiwan).
Xenocera ruficollis : Borchmann 1936: 117 (China: Taiwan); Sasaji 1986: 9 (China: Taiwan); Masumoto 1988: 46 (China: Taiwan); Chen 1997: 748 (China: Zhejiang, Fujian, Hubei, Chongqing, Taiwan).
Xenocerogria ruficollis : Merkl 2007: 270; Merkl 2008: 116 (China: Fujian, Taiwan).
Xenocera xanthisma Chen 2002: 178 (type locality: China: Fujian, Hunan; type depository: SWU). **syn. n.**

#### Redescription.

Body length 5–8 mm. Body, including antennae and legs, black, except brownish red prothorax and scutellum. Teneral specimens may have elytra reddish brown or whole body tending to be paler.

Male (Fig. 2). Head rounded, interocular distance 0.75× as wide as eye diameter; preorbital swelling elevated and glabrous; frons distinctly impressed, densely and coarsely punctate. Eyes reniform, slightly bulging, genal canthus encroaching to 0.7× eye width. Antennae (Figs 3–4) surpassing base of elytra when directed backwards, gradually broadening toward apex, antennomere 1 subglobular, 0.3× as long as distance between antennal insertions, antennomere 2 small, shorter than 1, antennomere 3 longer than 4 and 2, and about 2× longer than wide, antennomeres 5 to 9 short, trapezoidal, antennomere 10 strongly transverse, 2× as wide as long, antennomeres 5 to 10 glabrous in external side of ventral surface, antennomere 11 enormous, as long as combined length of 6 preceding antennomeres, as wide as antennomere 10, subparallel-sided, slightly curved, concave ventrally.

**Figures 1–6.**
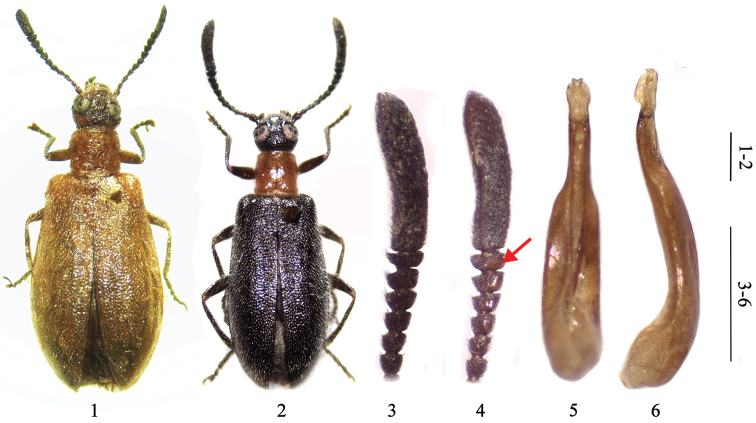
*Xenocerogria ruficollis* (Borchmann, 1912): **1** female, dorsal habitus **2** male, dorsal habitus **3** male antenna, dorsal view **4** male antenna, ventral view (arrow indicating glabrous part in external side) **5** aedeagus, ventral view **6** aedeagus, lateral view. Scale = 1 mm.

Pronotum subequal in length and width, barely constricted behind middle, maximum width just before middle; anterior and posterior angles rounded; disc with two indistinct oblique depressions before base; surface finely but densely punctate, punctures separated by interspaces of 0.3 to 0.5 puncture diameter on disc, tending to be subcontiguous toward lateral portions; disc with a small ill-defined impunctate spot at middle before base.

Elytra elongate, barely dilated posteriorly, widest at apical 1/3, about 4× as long as pronotum; punctation moderately dense, punctures separated by interspaces of 0.5 to 1 puncture diameter; interspaces slightly convex, forming short oblique or transverse wrinkles; dorsal pubescence consisting of short, semierect, sparsely set whitish hairs; humeral callosity separated from basal part of disc by indistinct impression; elytral margin visible in dorsal view except at humeral callosity; elytral epipleura densely punctate, parallel-sided from base to level of metacoxae, then gradually narrowing towards apex. Mesoventrite, mesepisternum, metepimeron, metepisternum finely and densely punctate; metaventrite very finely punctate, almost smooth, punctation becoming denser in lateral portions.

Legs narrow; apical 0.3 of middle and hind femora reaching beyond edge of elytra; fore and middle tibiae nearly straight, slightly shorter than femora, hind tibiae slightly curved, very weakly attenuated at middle, without visible denticulation. Tarsi simple.

Aedeagus with distal part of basale abruptly attenuating, much more slender than proximal part; apicale spoon-shaped, bifid at apex (Figs 5–6).

Female (Fig. 1). Larger than male. Head with interocular distance about 1.2× as wide as eye diameter. Preorbital swelling not developed. Antennomere 10 about 1.5× as wide as long; antennomere 11 shorter than combined length of 4 preceding antennomeres, but still broad. Elytra broader and more widening posteriorly. Legs shorter.

#### Type material examined.

**Figures 7–11.**
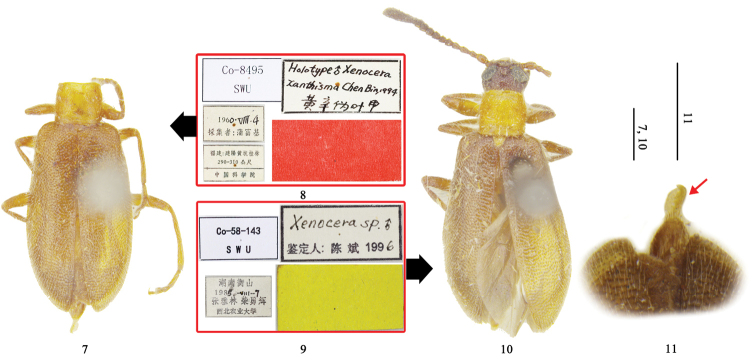
Types of *Xenocera xanthisma* Chen, 2002: **7** holotype, dorsal view **8** holotype, labels **9** paratype, labels **10** paratype, dorsal view **11** holotype, arrow indicating apex of aedeagus. Scale = 1 mm.

**Holotype** of *Xenocera xanthisma* Chen, 2002, male, (SWU, Fig. 7), pinned (head missing, but aedeagus visible (Fig. 11)), labelled (Fig. 8) as follows: 1) Co-8495 SWU [printed on white paper]; 2) 1960.VIII.4 collector: Fu-Ji Pu [first three numbers of year printed, last number of year, month and day handwritten, collector printed in Chinese on white paper]; 3) Fujian: Jianyang: Huangkeng: Guilin, 290–310 m, Chinese Academy of Science [printed on white label in Chinese]; 4) Holotype ♂ *Xenocera xanthisma* Chen Bin, 1994 [and the name of *Xenocera xanthisma* in Chinese, all handwritten on white paper]; 5) [red paper without text]. **Paratype** of *Xenocera xanthisma*, male (SWU, Fig. 10), labelled (Fig. 9) as follows: 1) Co-58-143 SWU [printed on white paper]; 2) Hunan: Hengshan, 1985.VII.7, Ya-Lin Zhang, Yong-Hui Cai, Northwestern Agricultural University [the last number of year, month and day handwritten, others printed in Chinese on white paper]; 3) *Xenocera* sp. ♂ det.: Chen Bin 1996 [“*Xenocera* sp.”, male symbol and the last number of year handwritten, others printed on white paper with black border]; 4) [yellow paper without text].

#### Other materials examined.

**Figures 12–13.**
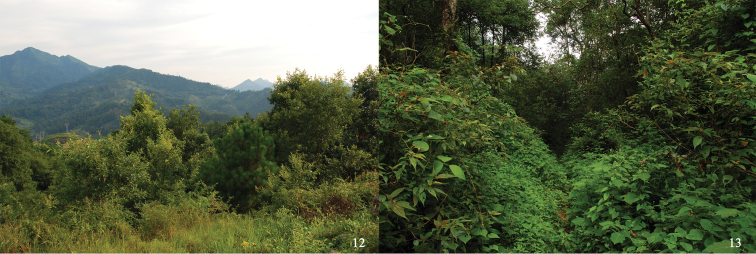
Habitats of *Xenocerogria ruficollis* (Borchmann, 1912): **12** Mt. Xiaogaoshan, Kaili, Guizhou **13** Maolan, Guizhou.

**China: Jiangsu:** 6 ♂♂ (CQNU), 1 ♂ (QCCC): Mt. Zijinshan, Nanjing, 27.V.2012, Hao Xu and Jian-Yue Qiu leg. **Fujian:** 1 ♂, 1 ♀ (HNHM): Shaowu env., 13–16.VI.1991, Nikodým & Červenka leg. **Guangxi:** 1 ♂ (MHBU): Bapen, Fusui, 17–18.VIII.2004, Yang Yu and Yi-Bin Ba leg.; 1 ♂ (MHBU): Luocheng, 21.VII.2006, Fu-Ming Shi and Shao-Li Mao leg. **Guizhou: ** 2 ♂♂ (MHBU): Sanchahe River, Libo, 29–31.VII.2010, Yong Zhou and Yi-Ping Niu leg.; 1 ♀ (CQNU): Maolan National Nature Reserve (Fig. 13), Libo County, 10.VIII.2012, Hao Xu and Jian-Yue Qiu leg.; 5 ♂♂ (CQNU), 1 ♂ (QCCC): Mt. Xiaogaoshan, Kaili (Fig. 12), 27.V.2013, Hao Xu and Jian-Yue Qiu leg. **Taiwan:** 1 (HNHM): Alishan, Chiayi Hsien, 22–25.VI.1974, M. Owada leg.; 4 (HNHM): Shanmei, Chiayi Hsien, 600 m, 2.V.1977, J. & S. Klapperich leg.; 2 (HNHM): Taihorinsho (= Talin), Chiayi Hsien, VIII.1909, H. Sauter leg.; 1 (HNHM): FuYuan Forest Recreation Area, Hualien Hsien, 28.V.1997, C. W. & L. B. O’Brien leg.; 1 (CCLT): Hohuenshan, Hualien Hsien, 13.VIII.2000, Chi-Feng Lee leg.; 1 (CCLT): Tienshiang, date and collector unknown; 3 (HNHM): Duona, Kaohsiung Hsien, 15.II.1993, M. L. Jeng leg.; 1 (HNHM): Liukuei, Kaohsiung Hsien, 29.IV.1970, Y. Kiyoyama leg.; 7 (HNHM): Kosempo (= Chiahsien), Kaohsiung Hsien, XI.1908 and IV.1909, H. Sauter leg.; 1 (CKMT): Paolai, Kaohsiung Hsien, 19.V.1975, K. Akiyama leg.; 2 (CKMT): Shanping, Kaohsiung Hsien, 27.IV.1981, S. Tsuyuki leg.; 1 (CKMT): Shanping, Kaohsiung Hsien, 1–2.V.1986, K. Masumoto leg.; 10 (HNHM): Shanping, Kaohsiung Hsien, 640 m, 23–31.III.1988, J. Rawlins & C. Young leg.; 33 (CMNH, HNHM): same locality and collectors, 1.IV.–20.V.1988; 3 (HNHM): Shanping Forest Recreation Area, near Liukuei, 22°58’16”N, 120°41’15”E, Kaohsiung Hsien, at light and swept & singled, 19–21.XI.2002, L. Ronkay & O. Merkl leg.; 17 (HNHM): Shanping LTER Site, near Liukuei, Kaohsiung Hsien, UV light trap, 1.IV.2003, L. Papp & M. Földvári leg.; 1 (HNHM): Shanping LTER Site, near Liukuei, along a creek, Kaohsiung Hsien, 1–2.IV.2003, L. Papp & M. Földvári leg.; 1 (HNHM): Takao (= Kaohsiung), Kaohsiung Hsien, 11.VII.1907, H. Sauter leg.; 1 (CMNH): Chungshin, Nantou Hsien, 30.V.1987, Chen Young leg.; 1 (HNHM): Fuhosho (= Wucheng), Nantou Hsien, VII.1909, H. Sauter leg.; 2 (NSMT): Hori, Nantou Hsien, 26.IV.1929, K. Sato leg.; 2 (HNHM): Hueisun, Nantou Hsien, 17.IV.1993, W. I. Chow leg.; 19 (HNHM): Huisun Forest Area, 15 km N of Puli, Nantou Hsien, 500 m, at light, 12–13. IV. 1997, G. Csorba & L. Ronkay leg.; 1 (HNHM): Huisun Forest Recreation Area, Nantou Hsien, 22. V. 1997, C. W. & L. B. O’Brien leg.; 1 (CKMT): Kuantoushan, Nantou Hsien, 18.VI.1993, Luo Chinchi leg; 1 (NSMT): Lienhuachi, Nantou Hsien, 750 m, 14–16.III.1980, T. Shimomura leg.; 1 (CKMT): same locality and collector, 16–17.III.1980; 2, Lienhuachi, Nantou Hsien, 27.VI.1998, K. Akiyama leg.; 1 (NSMT): Lushan, Nantou Hsien, 8.V.1975, K. Akiyama leg.; 2 (HNHM): 4 km above Lushan, Nantou Hsien, 18.V.1997, C. W. & L. B. O’Brien leg.; 21 (HNHM): Mong Gwu, 14 km E of Puli, 24°1.367'N, 121°5.063'E, Nantou Hsien, 850 m, swept from vegetation, 20.IV.2002, D. Anstine, Gy. Fábián & O. Merkl leg.; 3 (CKMT): Nanshanchi, Nantou Hsien, 16.V.1971, K. Sakai leg.; 1 (HNHM): Nanshanchi, Nantou Hsien, 27.VII.1972, K. Masumoto leg.; 6 (HNHM): same locality and collector, 23 to 29.IV.1974; 1 (CKMT): same locality and collector, 29.IV.1994; 1 (CKMT): Nanshanchi, Nantou Hsien, 30.III.1972, Y. Miyake leg.; 1 (CKMT): Nanshanchi, Nantou Hsien, 29.IV.1973, S. Tsuyuki leg.; 1 (CKMT): Nanshanchi, Nantou Hsien, 5.V.1979, K. Emoto leg.; 5 (CKMT): Nanshanchi, Nantou Hsien, 3–15.IV.1986, M. Ohara leg.; 1 (CKMT): Nanshanchi, Nantou Hsien, 28.VII.1990, collector unknown; 1 (CKMT): Shizitou, Nantou Hsien, 7.V.1992, Luo Chinchi leg.; 4 (CCLT): Tungpu, Nantou Hsien, 13.IX.2001, C.-F. Lee; 1 (CKMT): Wushe, Nantou Hsien, 4.V.1979, S. Tsuyuki leg.; 1 (CKMT): Fenshuiling, Pingtung Hsien, 14.V.1996, S. Tsuyuki leg.; 1 (HNHM): Kenting National Park, Botanical Garden, Pingtung Hsien, 4–6.X.2000, L. Papp, L. Peregovits & L. Ronkay leg.; 2 (HNHM): Nanjensan, Pingtung Hsien, 13.III.1993, W. I. Chow leg.; 2 (HNHM): Taipei, Taipei City, 24.IX.2000, L. Papp, L. Peregovits & L. Ronkay leg.; 2 (NSMT): Yangmingshan, Taipei City, 25.V.1965, K. Morimoto leg.; 1 (HNHM): Yangmingsan, Taipei City, 15.IX.1970, Y. Kiyoyama leg.; 3 (HNHM): Guanyinshan, Taipei Hsien, 500 m, singled, 15.XI.2002, O. Merkl leg.; 1 (HNHM): Haeng-Lu Dyi, Taipei Hsien, swept, 2.IV.2002, Gy. Fábián & O. Merkl leg.; 15 (HNHM): same locality and collectors, around lights, 2–21.IV.2002; 1 (NSMT): Wulai, Taipei Hsien, 27.V.1965, K. Morimoto leg.; 2, Wulai, Taipei Hsien, 200 m, 3.IV.1977, J. & S. Klapperich leg.; 1 (CKMT): Chihpen, Taitung Hsien, 27.IV.1986, K. Masumoto leg.; 1 (HNHM): 5 km S Chinglun, Taitung Hsien, 30.V.1997, C. W. & L. B. O’Brien leg.; 3 (CKMT): Paling, Taoyuan Hsien, 28–29.IV.1979, S. Tsuyuki leg.; 1 (HNHM): Paling, Taoyuan Hsien, 25.IV.1982, N. Ohbayashi leg.; 1 (CCLT): Paling, Taoyuan Hsien, 23.V.1999, C.-F. Lee leg.; 1 (MHNG): Upper Paling, Taoyuan Hsien, 1200 m, 18.IV.1990, A. Smetana leg.

#### Distribution.

China: Jiangsu (new record), Zhejiang, Fujian, Hubei, Guangxi (new record), Chongqing, Guizhou (new record), Taiwan. In Taiwan, *Xenocerogria ruficollis* is one of the most common species of Lagriini, especially in the *Machilus*-*Castanopsis* zone, including disturbed places and secondary growth. Specimens are known from the lower *Quercus* zone, too. (The altitudinal vegetation zones of Taiwan see Su 1984).

#### Remarks.

*Xenocera xanthisma* was described by Chen (2002) on the basis of three males and one female from Fujian and Hunan. However, the original description was not mentioned in the Zoological Record, therefore this species was unknown to other coleopterists, and was not included in the Catalogue of Palaearctic Coleoptera (Merkl 2008). The type series was deposited in SWU, but at the moment only the male holotype and one male paratype are found there. The holotype is in bad condition with its head missing, but fortunately, the aedeagus is visible. Chen (2002) indicated “yellow color and curve[d] terminal antennomere” as diagnostic to *Xenocera xanthisma*. However, “curve[d] terminal antennomere” is also characteristic to *Xenocerogria ruficollis*. “Yellow color” is typical to teneral individuals of *Xenocerogria ruficollis* that are pale yellowish brown with head and antennae somewhat darker. Moreover, the aedeagus of the holotype is identical with that of *Xenocerogria ruficollis*. Therefore we propose *Xenocera xanthisma* as a junior subjective synonym of *Xenocerogria ruficollis* (Borchmann, 1912).

### Xenocerogria feai

Taxon classificationAnimaliaColeopteraTenebrionidae

(Borchmann, 1911)

**Figures 14–17.**
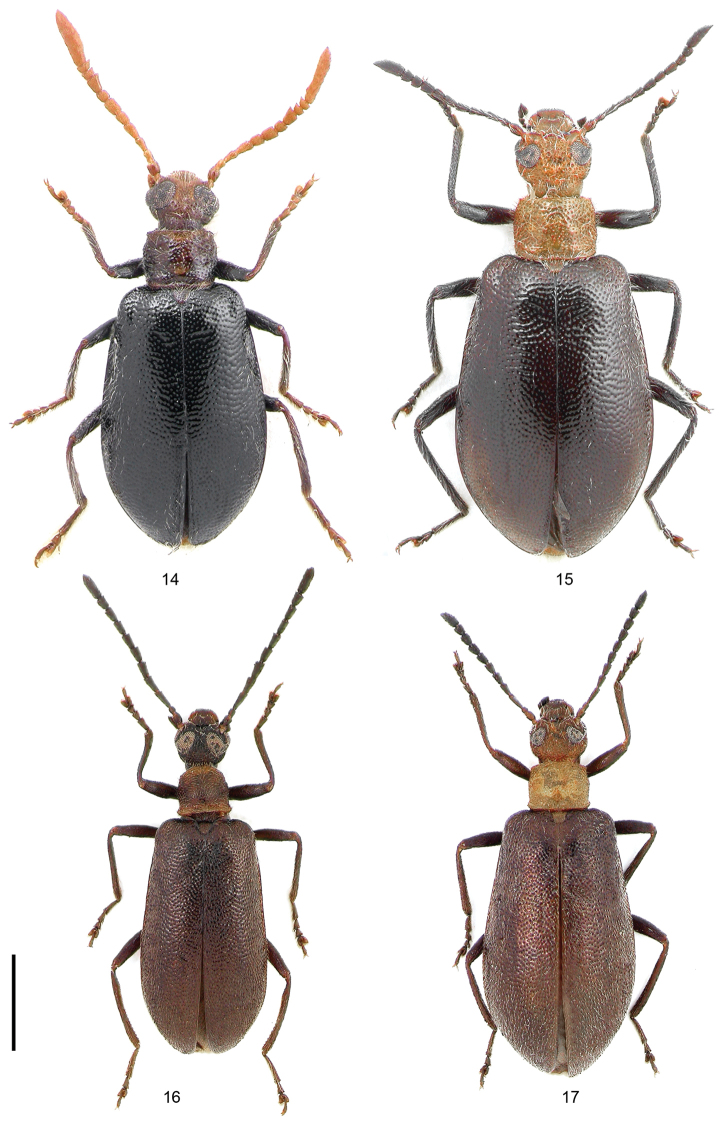
Dorsal habitus: **14** *Xenocerogria feai*, male **15** *Xenocerogria feai*, female **16** *Lagria ignota*, male **17** *Lagria ignota*, female. Scale = 2 mm.

**Figures 18–25.**
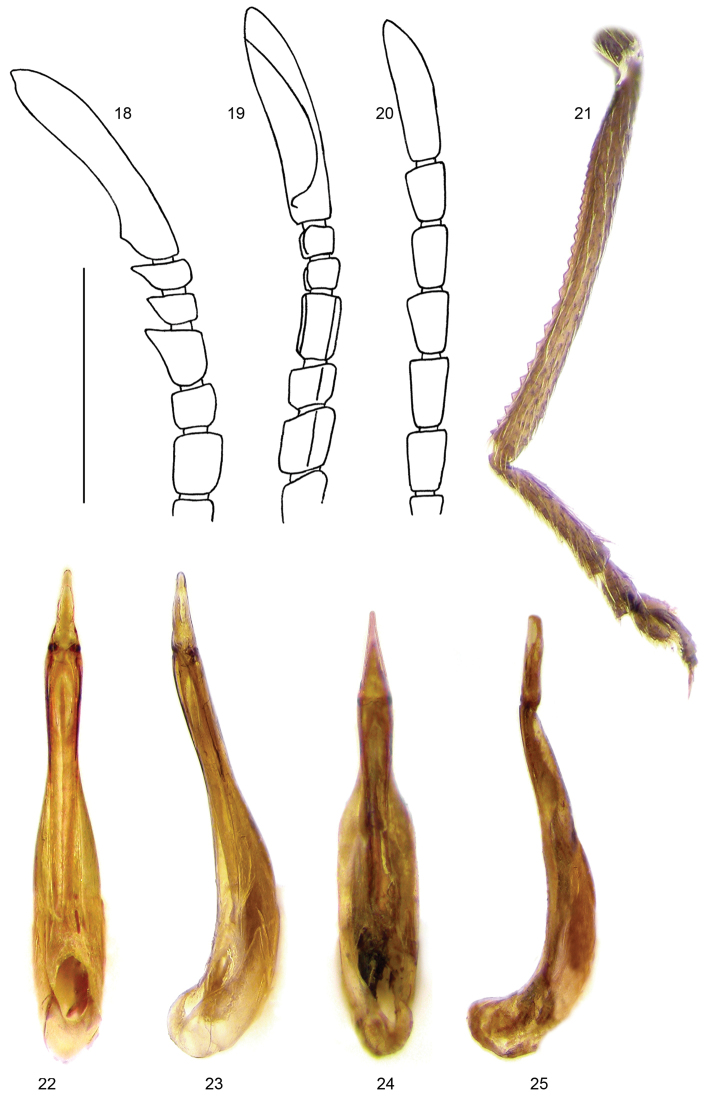
Distal six male antennomeres: **18** *Xenocerogria feai*, lateral view **19** *Xenocerogria feai*, ventrolateral view **20** *Lagria ignota*, lateral view **21** *Lagria ignota*, male right hind tibia **22–25** Aedeagus: **22** *Xenocerogria feai*, ventral view **23** *Xenocerogria feai*, lateral view **24** *Lagria ignota*, ventral view **25** *Lagria ignota*, lateral view. Scale = 1 mm.

Figs 14–15 , 18–19 , 22–23

Lagriocera feae Borchmann, 1909: 209 (type locality: Burma, Carin Chebà. type depository: MSNG).
Xenocera feai : Borchmann 1936: 117.
Xenocerogria feai : Merkl 2007: 270 (China: Yunnan; Burma); Merkl 2008: 116.

#### Redescription.

Body length 5–10 mm. Body, including legs, black, except brownish red head, antennae, prothorax and scutellum. Brownish red parts sometimes darker brown to black.

Male (Fig. 14). Head rounded, interocular distance 0.75× as wide as eye diameter; preorbital swelling slightly convex and glabrous; frons distinctly impressed, sparsely and coarsely punctate. Eyes reniform, moderately bulging, genal canthus encroaching to 0.6× eye width. Antennae (Figs 18–19) surpassing base of elytra when directed backwards, gradually broadening toward apex, antennomere 1 subglobular, 0.3× as long as distance between antennal insertions, antennomere 2 small, shorter than 1, antennomere 3 longer than 2 but shorter than 4, 5 subquadrate, shorter than 4, 6 longer and wider than 5 and 7, 7 slightly transverse, 8 twice longer than 7, anterior inner angle slightly produced, 9 and 10 strongly transverse, inner anterior angle almost dentiform; inner surface of antennomeres 5 to 10 flattened, smooth, glabrous, bordered with fine carinae; antennomere 11 as long as combined length of 4 preceding antennomeres, as wide as 10, subparallel-sided, strongly concave ventrally, inner margin of concavity bordered with carina forming sharp angulation in basal quarter.

Pronotum moderately transverse, maximum width at middle, anterior and posterior angles rounded; disc with four indistinct transverse lateral impressions; surface coarsely, sparsely and irregularly punctate, punctures separated by interspaces of 0.3 to 1.5 puncture diameter on disc, tending to be subcontiguous toward lateral portions; longitudinal midline with fine, obsolete carina.

Elytra elongate, moderately dilated posteriorly, widest at apical 1/3, about 5× as long as pronotum; punctation moderately dense, punctures separated by interspaces of 0.5 to 1 puncture diameter; interspaces slightly convex, forming short oblique or transverse wrinkles; dorsal pubescence consisting of long, erect, sparsely set whitish hairs; humeral callosity separated from basal part of disc by distinct impression; elytral margin visible in dorsal view except at humeral callosity; elytral epipleura densely punctate, parallel-sided from base to level of metacoxae, then gradually narrowing towards apex. Mesoventrite, mesepisternum, metepimeron, metepisternum finely and sparsely punctate; metaventrite very finely punctate, almost smooth, punctation becoming denser in lateral portions.

Legs narrow; apical 0.3 of middle and hind femora reaching beyond edge of elytra; fore and middle tibiae nearly straight, slightly shorter than femora, hind tibiae straight, with sparse tuft of short and fine hairs before middle, without visible denticulation. Tarsi simple.

Aedeagus with distal part of basale narrow, gradually attenuating, apicale narrow, pointed (Figs 22–23).

Female (Fig. 15). Larger than male. Head with interocular distance about 1.2× as wide as eye diameter. Preorbital swelling not developed. Antennomeres not modified, 5 to 10 without flattened and smooth inner surface and produced inner anterior angle; antennomere 9 quadrate, 10 slightly transverse; antennomere 11 not concave, shorter than combined length of 2 preceding antennomeres. Elytra broader and more widening posteriorly. Legs shorter, hind tibiae without sparse tuft.

#### Type material examined.

One syntype, female (MSNG, examined by O. Merkl in 1990), labelled as follows: 1) Carin Chebà 900–1100 m L. Fea V XII-88 [printed in black frame on white paper]; 2) Typus [printed with red in red frame on white paper]; 3) Feae Borch. [printed on white paper with black frame]; 4) Lagriocera Feae m. [Borchmann’s handwriting on white paper in black frame]; 5) SYNTYPUS Lagriocera feae Borchmann 1911 (1909) [printed and handwritten on pink paper]; 6) Museo Civico di Genova [printed on white paper]. One syntype, male (MSNG, examined by O. Merkl in 1990), labelled as follows: 1) Carin Chebà 900–1100 m L. Fea V XII-88 [printed in black frame on white paper]; 2) SYNTYPUS Lagriocera feae Borchmann 1911 (1909) [printed and handwritten on pink paper]; 6) Museo Civico di Genova [printed on white paper].

#### Other materials examined.

**China: Yunnan:** 1 ♂, 1 ♀ (CSBC), 5 ♂♂, 9 ♀♀ (HNHM): Jinghong, 10–14.VII.1990, S. Bečvář leg.

#### Distribution.

China: Yunnan; Burma.

### Lagria ignota

Taxon classificationAnimaliaColeopteraTenebrionidae

(Borchmann, 1941) comb. n.

Figs 16–17 , 20–21 , 24–25

Xenocera ignota Borchmann, 1941: 26 (type locality: China, Fujian, Kuatun. type depository: ZFMK).
Xenocerogria ignota : Merkl 2007: 270; Merkl 2008: 116 (China: Fujian; Oriental realm).

#### Redescription.

Body length 5–8 mm. Body, including antennae and legs, black, except brownish red head, prothorax and scutellum. Brownish red parts sometimes darker brown to black.

Male (Fig. 16). Head rounded, interocular distance 0.5× as wide as eye diameter; preorbital swelling convex and glabrous; frons weakly impressed, densely and coarsely punctate. Eyes reniform, moderately bulging, genal canthus encroaching to 0.75× eye width. Antennae (Fig. 20) surpassing middle coxae when directed backwards, not broadening toward apex, antennomere 1 slightly longer than wide, 0.4× as long as distance between antennal insertions, antennomere 2 small, shorter than half of 1, antennomere 3 nearly 3× longer than 2 and 1.5× longer than 4 to 7 subequal in length, more than 2× longer than wide, 8 and 9 slightly narrower than preceding ones but still 2× longer than wide, 10 1.5× longer than wide, 11 about as long as 9 and 10 combined; none of apical antennomeres having modifications.

Pronotum moderately transverse, maximum width at middle, anterior and posterior angles rounded; disc with four indistinct transverse lateral impressions; surface coarsely, densely and irregularly punctate, punctures separated by interspaces of 0.3 to 0.5 puncture diameter on disc, tending to be subcontiguous mainly toward lateral portions; longitudinal midline with hardly discernible carina.

Elytra elongate, barely dilated posteriorly, widest at apical 1/5, about 5× as long as pronotum; punctation moderately dense, punctures separated by interspaces of 0.5 to 1 puncture diameter; interspaces slightly convex, forming short oblique or transverse wrinkles, mainly in basal 2/3; dorsal pubescence consisting of short, decumbent, sparsely set whitish hairs; humeral callosity separated from basal part of disc by vague impression; elytral margin visible in dorsal view except at humeral callosity; elytral epipleura sparsely punctate, parallel-sided from base to level of metacoxae, then gradually narrowing towards apex. Mesoventrite, mesepisternum, metepimeron, metepisternum finely and sparsely punctate; metaventrite very finely punctate, almost smooth, punctation becoming denser in lateral portions.

Legs narrow; apical half of middle and hind femora reaching beyond edge of elytra; fore and middle tibiae nearly straight, slightly shorter than femora, hind tibiae (Fig. 21) weakly curved, slightly dilated at basal ¼, inner margin between dilatation and apex with fine denticulation. Tarsi simple.

Aedeagus with distal part of basale broad, then abruptly attenuating, apicale narrow, pointed (Figs 24–25).

Female (Fig. 17). Larger than male. Head with interocular distance about 1.2× as wide as eye diameter. Genal canthus encroaching to 0.85× eye width. Preorbital swelling not developed. Antennomeres 11 shorter 9 and 10 combined length. Elytra broader and more widening posteriorly. Legs shorter, hind tibiae straight, without denticulation.

#### Type material examined.

Lectotype, herewith designated, female (ZFMK), mounted on a card, left fore tarsus and middle right leg are missing, labelled as follows: 1) Kuatun (2300 m) 27,40n. Br. 117,40ö. L. J. Klapperich 4. 6. 1938 (Fukien) [printed on pale pink paper]; 2) Type [printed on dark pink paper with black frame]; 3) Xenocera ignota m. [Borchmann’s handwriting on white paper]; 4) MUSEUM KOENIG BONN [printed on orange paper]; 5) Lectotypus ♀ *Xenocera ignota* Borchmann, 1941, des. Y. Zhou, O. Merkl & B. Chen, 2014 [printed on red paper].

#### Other materials examined.

**China: Fujian:** 1 ♂ (ZFMK), 1 ♀ (HNHM): Kuatun [=Guadun, in Mt. Wuyishan], N 27°40’, E 117°40’, 11.IV.1938, L. J. Klapperich leg.; 1 ♂ (ZFMK): same locality and collector, 7.V.1938; 4 ♂♂, 1 ♀ (ZFMK), 3 ♂♂, 2 ♀♀ (HNHM): same locality and collector, 8.V.1938; 4 ♂♂, 1 ♀ (ZFMK), 2 ♂♂ (HNHM): same locality and collector, 11.V.1938; 7 ♂♂, 4 ♀♀ (ZFMK), 2 ♂ ♂ (HNHM): same locality and collector, 12.V.1938; 3 ♀♀ (ZFMK): same locality and collector, 13.V.1938; 2 ♂♂, 1 ♀ (ZFMK): same locality and collector, 19.V.1938; 1 ♀ (ZFMK): same locality and collector, 23.V.1938; 3 ♀♀ (ZFMK): same locality and collector, 24.V.1938; 1 ♂, 1 ♀ (ZFMK): same locality and collector, 26.V.1938; 1 ♀ (ZFMK): same locality and collector, 25.V.1938; 2 ♀♀ (ZFMK): same locality and collector, 30.V.1938; 1 ♀ (ZFMK): same locality and collector, 2.VI.1938; 1 ♂, 2 ♀♀ (ZFMK): same locality and collector, 4.VI.1938; 1 ♀ (ZFMK): same locality and collector, 6.VI.1938; 1 ♂ (ZFMK): same locality and collector, 8.VI.1938; 1 ♀ (ZFMK): same locality and collector, 14.VI.1938; 1 ♀ (ZFMK): same locality and collector, 15.VI.1938; 1 ♀ (ZFMK): same locality and collector, 20.VI.1938. **Vietnam: Vinh phu Province:** 1 (SMNS): 15–17.IV.1986, Tamdao, 80 km N of Hanoi, 900 m, collector unknown; 1 (SMNS): 19–21.IV.1986, same locality; 1 (SMNS), 1 (HNHM): 20.IV.1986, same locality; 1 (SMNS): 24–25.V.1985, same locality.

#### Distribution.

China: Fujian; Vietnam.

#### Remarks.

Borchmann (1941) described this species based on resemblance of the two female syntypes to *Xenocerogria ruficollis*. Male specimens were not available to him, although the long series collected by Klapperich in Fujian that included the two syntypes, contained several males as well. If he could have seen males, he would not have described the species in the genus *Xenocera*, because the male is unlike to that of the congeners: antennomeres 9 and 10 are not transverse, antennomere 11 is short and not concave ventrally, and the hind tibiae have distinctive serration. If it is accepted that *Xenocerogria* is defined as having enlarged, concave antennomere 11 and unmodified tibiae of males, the only plausible approach is to remove *Xenocerogria ignota* from this genus and transfer it to the composite genus of *Lagria*, for lack of a better place to put it.

## Supplementary Material

XML Treatment for Xenocerogria

XML Treatment for Xenocerogria ruficollis

XML Treatment for Xenocerogria feai

XML Treatment for Lagria ignota
